# Oxaliplatin-induced neurotoxicity involves TRPM8 in the mechanism of acute hypersensitivity to cold sensation

**DOI:** 10.1002/brb3.34

**Published:** 2012-01

**Authors:** Toru Kono, Machiko Satomi, Manabu Suno, Norihisa Kimura, Hirotaka Yamazaki, Hiroyuki Furukawa, Kazuo Matsubara

**Affiliations:** 1 Division of Gastroenterologic and General Surgery, Department of Surgery, Asahikawa Medical UniversityAsahikawa, Japan; 2 Division of Chemotherapy, Higashi-Asahikawa HospitalAsahikawa, Japan; 3 Department of Hospital Pharmacy and Pharmacology, Asahikawa Medical UniversityAsahikawa, Japan

**Keywords:** Menthol, neurotoxicity, oxaliplatin, transient receptor potential melastatin 8

## Abstract

Oxaliplatin-induced peripheral neurotoxicity (OPN) is commonly associated with peripheral hypersensitivity to cold sensations (CS) but the mechanism is unknown. We hypothesized that the transient receptor potential melastatin 8 (TRPM8), a putative cold and menthol receptor, contributes to oxaliplatin cold hypersensitivity. To determine whether the TRPM8 is involved in acute OPN, varying concentrations of menthol were topically applied to the tongues of healthy subjects (*n*= 40) and colorectal cancer patients (*n*= 36) before and after oxaliplatin administration. The minimum concentration of menthol to evoke CS at the menthol application site was determined as the CS detection threshold (CDT). In healthy subjects, the mean CDT was 0.068. Sex and age differences were not found in the CDT. In advanced colorectal cancer patients, the mean CDT significantly decreased from 0.067% to 0.028% (*P*= 0.0039) after the first course of oxaliplatin infusions, and this marked CS occurred in patients who had grade 1 or less neurotoxicity, and grade 2 neurotoxicity, but not in those with grade 3 neurotoxicity. Further, the mean baseline CDT in oxaliplatin-treated patients was significantly higher than that of chemotherapy-naïve patients and healthy subjects (0.151% vs. 0.066%, *P*= 0.0225), suggesting that acute sensory changes may be concealed by progressive abnormalities in sensory axons in severe neurotoxicity, and that TRPM8 is subject to desensitization on repeat stimulation. Our study demonstrates the feasibility of undertaking CDT test in a clinical setting to facilitate the identification of early neurotoxicity. Moreover, our results indicate potential TRPM8 involvement in acute OPN.

## Introduction

Oxaliplatin-induced peripheral neurotoxicity (OPN) is deleterious to patients both in terms of troublesome symptoms and the need to reduce or discontinue chemotherapy (Adelsberdger et al. 2000). Oxaliplatin, a third-generation platinum analog, causes a unique spectrum of acute peripheral nerve hyperexcitability that has not been observed in patients receiving other platinum chemotherapeutic agents. Conversely, chronic oxaliplatin treatment induces an axonal neuropathy that is similar to that observed with other platinum-based compounds (Lehky et al. 2004). In clinical studies, approximately 90% of oxaliplatin-treated patients experienced unique acute OPN, particularly cold-induced paresthesia that is usually triggered by cold exposure and begins in the hands or feet but sometimes occurs around the mouth or in the throat (Raymond et al. 1998a; Raymond et al. 1998b; Grothey, 2003; Ali 2010;). It is an acute transient syndrome that may begin during drug infusion or within minutes, hours, or 1–2 days after administration but is usually self-limiting, often disappearing within a few days (Gamelin et al. 2002, 2006).

Recently, a wide repertoire of sensory transduction molecules that convert external environmental stimuli into neural activity has been identified (Basbaum et al. 2009). For example, the transient receptor potential (TRP) family of ion channels are the primary detectors of thermal stimuli (Jordt et al. 2003), and TRP melastatin 8 (TRPM8) determines whether temperatures are considered cool or cold (McKemy et al. 2002; Peier et al. 2002; Daniels and McKemy 2007). However, to date, there is no evidence that TRPM8 is involved in the mechanisms of acute OPN.

Menthol, a potent TRPM8 agonist, has long been known to induce or intensify cold sensations by interacting with the peripheral cold receptor, TRPM8 (McKemy et al. 2002; Peier et al. 2002; Knowlton et al. 2010). The tongue is a well-characterized sensory organ, and TRPM8 is present in sensory lingual nerve fibers that mainly project from the trigeminal ganglion where they function as cold and menthol receptors on the tongue (Abe et al. 2005).

On the basis of these observations, we hypothesized that TRPM8 is involved in the mechanisms of acute OPN, especially marked sensitivity to cold. We tested this hypothesis by topically applying varying concentrations of menthol, a TRPM8 agonist, to the patients’ tongue before and after oxaliplatin infusions to determine their sensitivity to cold sensation. The minimum concentration of menthol to evoke cold sensation (CS) at the menthol application site was determined as the cold sensation detection threshold (CDT).

The conventional clinical grading system was used to assess the severity of neurotoxicity in relation to CDT. Patients also completed self-report ratings of their sensitivity to cold sensation, and the results of these objective and subjective findings were compared.

## Materials and Methods

### Subjects and treatment regimen

A total of 76 subjects were enrolled in this study: 40 healthy subjects (24 women, 16 men; median age, 54 years; range, 22–85) and 36 patients (22 women, 14 men; median age, 57 years; range, 33–80) with advanced-stage colorectal cancer who received standard oxaliplatin in combination with infusional 5-fluorouracil/leucovorin (FOLFOX) as a first-line treatment. In the FOLFOX regimens, oxaliplatin (modified FOLFOX 6, 85 mg/m^2^) was given intravenously over 2 h on day 1 in conjunction with leucovorin (200 mg/m^2^) and followed by a 5-fluorouracil (5-FU) bolus injection (400 mg/m^2^), repeated every 2 weeks. A continuous 24-h infusion of 5-FU (600 mg/m^2^) was given over days 1 and 2. On day 2, leucovorin (200 mg/m^2^; over 2 h) and 5-FU bolus (400 mg/m^2^) were given intravenously.

The subjects did not consume any spicy food 1 day prior to testing. They were also asked to refrain from eating, drinking, chewing gum, brushing their teeth, and using mouthwash for 2 h before testing, and we verified that the participants had observed these restrictions at the beginning of each session.

The present study was conducted in accordance with the Declaration of Helsinki for the care for human studies adopted by the Ethics Committee of Higashi-Asahikawa Hospital. All patients provided written informed consent.

### Assessment of menthol in experiments 1 and 2

**Figure 1:**
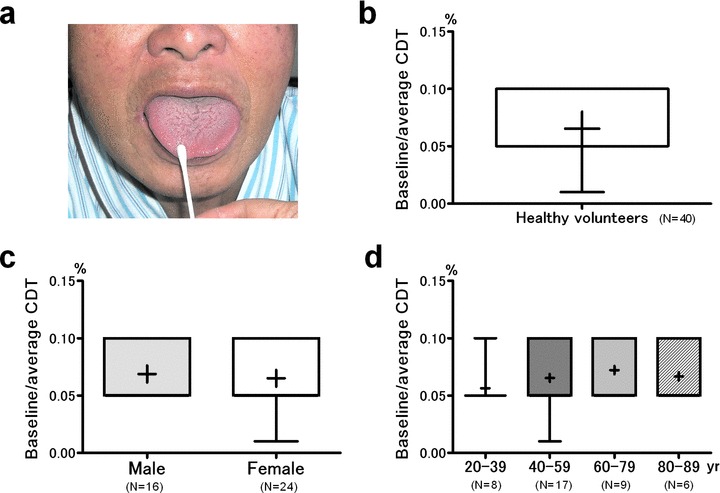
Effects of menthol on cold sensation and the detection threshold in healthy human subjects. (a) The menthol solution was topically applied with a cotton swab to the dorsal anterior tongue. (b) The mean baseline cold sensation detection thresholds (CDTs) in healthy human volunteers (*n*= 40) were 0.01% (1 of 40 subjects), 0.05% (26 of 40), and 0.1% (13 of 40). The overall mean CDT was 0.068 ± 0.026% (mean ± SD). (c) Significant sex difference in mean baseline CDT was not found. (d) Significant age difference in mean baseline CDT was not found. Cross is the mean of CDT.

A solution of 5% L-menthol (from dry crystals; MERCK, Tokyo, Japan) was prepared in warm distilled water (41°C) at the time of application, and this solution was further diluted in warm distilled water to yield menthol solutions of 0.005%, 0.01%, 0.05%, 0.1%, 0.5%, and 1% (0.32 mM, 0.64 mM, 3.2 mM, 6.4 mM, 32 mM, and 64 mM, respectively). These solutions were topically applied with a cotton swab to the dorsal anterior tongue in two experiments (Fig. 1a). In experiment 1, the six different menthol solutions were administered to healthy subjects and patients with colon cancer prior to oxaliplatin exposure, and their subjective ratings of cold sensitivity were recorded. In experiment 2, patients were examined for alterations in the menthol-induced cold sensations before and 5–6 h after the patients receiving individual oxaliplatin infusions. The menthol concentrations used in this study were based on a previous human study (Albin et al. 2008). The vehicle control (warmed distilled water) was applied in the same manner.

Both experiments were performed in a room maintained at a constant temperature (22 ± 1°C) and a relative humidity of 55 ± 5%. The menthol testing was performed by two investigators (TK and MS) on all participants. Neither the individuals nor the investigator were aware of whether menthol or the vehicle was applied first because the substances were encoded by a technical assistant.

### Cold sensations and Cold sensation detection threshold

The highest and lowest concentrations of the menthol solutions were set at 1% and 0.005%, respectively. Starting at the lowest and increasing to the highest menthol concentration, the solutions were applied with an interstimulus interval of 10 sec. For each stimulus, the subject was instructed to push a button as soon as he or she detected a CS (CDT). The CDT was considered the minimum menthol concentration. When no threshold was obtained, the highest concentration tested (1%) was entered as the threshold value.

### Assessment of neurotoxicity

The National Cancer Institute Common Terminology Criteria for Adverse Events (CTCAE) version 3.0 was used to evaluate the severity of neurotoxicity: grade 1 (mild), loss of deep tendon reflexes or paresthesia not interfering with function; grade 2 (moderate), sensory alteration or paresthesia interfering with function but not activities of daily living; grade 3 (severe), sensory alteration or paresthesia interfering with activities of daily living; and grade 4, disabling (Trotti et al. 2003).

### Statistics

The effects of oxaliplatin were analyzed by the nonparametric Wilcoxon *t*-test for paired samples. In all of the statistical analyses, significance was determined using an alpha level of 0.05. All statistical procedures were performed using the IBM-SPSS software package version 18.0J for Windows (Tokyo, Japan) and the GraphPad Prism 4 statistics program (GraphPad Software, Inc., San Diego, CA).

## Results

### Effects of menthol on CS and CDT in healthy human subjects and patients with colon cancer (experiment 1)

All subjects noticed a significant feeling of coldness at the menthol application site. The CS occurred within the first 3 sec, reached an intensity plateau at approximately 5 sec and then disappeared within 10 sec. The intensity of the CS increased in a dose-dependent manner. None of the subjects experienced a CS when the vehicle control was applied. The mean baseline CDTs in healthy human volunteers were 0.01% (1 of 40 subjects), 0.05% (26 of 40), and 0.1% (13 of 40). The mean CDT was 0.068 ± 0.026% (SD) (Fig. 1b). To assess reproducibility, 40 healthy subjects were retested, and their CDTs were found not to differ significantly from the previous testing. Significant sex and age differences in mean baseline CDTs were not found as well (Fig. 1c and d). No serious adverse events occurred during the study and all doses of menthol were well tolerated.

The mean CDT in patients with colon cancer who had never received any chemotherapy was 0.067 ± 0.025% (*n*= 12). No significant difference in mean baseline CDT was observed between healthy subjects and patients with colon cancer. In addition, no serious adverse events occurred during the study and all doses of menthol were well tolerated.

### Changes in the CDT before and after oxaliplatin administration (experiment 2)

**Figure 2:**
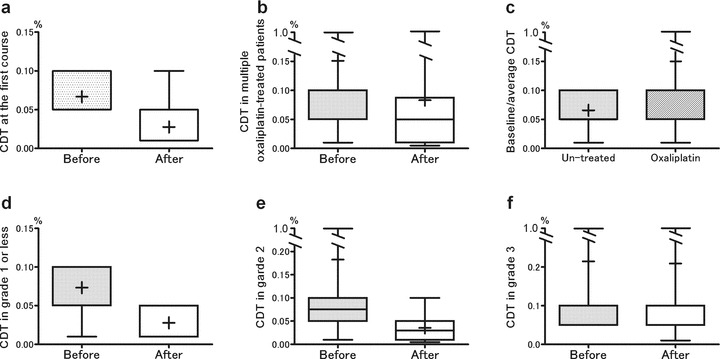
Changes in the cold sensation detection threshold (CDT) before and after oxaliplatin administration. (a) The CDT was determined by applying menthol before and after the first oxaliplatin administration. The CDT significantly decreased from 0.067 ± 0.025% (mean ± SD) to 0.028 ± 0.029% (*n*= 12, *P*= 0.0025). (b) Changes in the CDT before and after oxaliplatin administration in patients previously treated with oxaliplatin. The CDT significantly decreased from 0.151 ± 0.263% to 0.083 ± 0.198% (*n*= 24, *P*= 0.0004). (c) The mean baseline CDT was significantly higher in patients previously treated with oxaliplatin (*n*= 24) than in untreated subjects (*n*= 52) (0.151% vs. 0.066%, *P*= 0.0225). (d) The CDT was measured before and after oxaliplatin was administered to patients who had grade 1 or less neurotoxicity. The CDT significantly decreased from 0.073 ± 0.034% to 0.028 ± 0.021% (*n*= 9, *P*= 0.0126). (e) The CDT was measured before and after oxaliplatin was administered to patients who had grade 2 neurotoxicity. There was no significant difference in the CDTs (*n*= 8; before, 0.183 ± 0.332%; after, 0.036 ± 0.033%; *P*= 0.022). (f) The CDT was obtained before and after oxaliplatin was administered to patients who had grade 3 neurotoxicity. There was no significant difference in the CDTs (*n*= 7; before, 0.214 ± 0.347%; after, 0.209 ± 0.351%; *P*= 1.0). Cross is the mean of CDT.

Figure 2a shows the CDTs that were obtained before and after the first oxaliplatin administration in patients who had never received chemotherapy. All but one patient were hypersensitive to menthol as indicated by a significant decrease in the CDT from 0.067 ± 0.025% to 0.028 ± 0.029% (*n*= 12, *P*= 0.0025). The CDTs were also measured before and after oxaliplatin administration in patients who had previously received oxaliplatin (*n*= 24, median, 330 mg/m^2^; range, 85 − 2450 mg/m^2^). Under these conditions, the CDT significantly decreased from 0.151 ± 0.263% to 0.083 ± 0.198% (*n*= 24, *P*= 0.0004) (Fig. 2b). Taken together, these findings show that the mean baseline CDT was significantly higher in patients previously treated with oxaliplatin (*n*= 24) than in untreated subjects (*n*= 52) (0.151% vs. 0.066%, *P*= 0.0225).

When the relationship between the CDTs and the CTCAE neurotoxicity ratings in oxaliplatin-treated patients was evaluated, the CDTs were found to be significantly decreased in patients who had grade 1 or less neurotoxicity (from 0.073% to 0.028%) (*n*= 9, *P*= 0.0126) (Fig. 2d), and grade 2 (from 0.183% to 0.036%) (*n*= 8, *P*= 0.022) (Fig. 2e), but not in those with grade 3 neurotoxicity (from 0.214% to 0.209%) (*n*= 7, *P*= 1.0) (Fig. 2f).

## Discussion

Our results indicate a potential correlation between TRPM8 activity and OPN, especially in acute hypersensitivity to CS, and that acute changes in CDT may facilitate the identification of early OPN. In chemotherapy-naïve patients, significant sensitivity to topical menthol developed after the first oxaliplatin infusion, suggesting that oxaliplatin had indeed induced cold hypersensitivity. In contrast, patients with previous oxaliplatin exposure showed reduced cold hypersensitivity. With regard to the relationship between the CDT and neurotoxicity grade, we found that mild or moderate neurotoxicity was associated with significant changes in the CDT, while severe neurotoxicity was not associated with marked changes in the CDT. Whether the CDT remains unaltered in oxaliplatin-treated patients who do not develop OPN despite chronic oxaliplatin exposure requires further investigation. Nonetheless, these findings suggest that the CDT is a sensitive marker of early oxaliplatin-induced sensory disturbances.

Menthol activates the cold-transducing Ca^2+^ ion channel TRPM8 and increases cold-evoked currents (McKemy et al. 2002; Peier et al. 2002), and TRPM8 is naturally expressed sensory neurons (Reid et al. 2002; Abe et al. 2005; Kobayashi et al. 2005; Madrid et al. 2006). These TRPM8-expressing sensory neurons project into the superficial laminae of the spinal cord dorsal horn (Dhaka et al. 2008; Wrigley et al. 2009) that contains cold-sensitive neurons that project into the spinothalamic tract (Craig and Dostrovsky 2001). Thus, the cold-induced paresthesias after oxaliplatin administration that were accentuated by menthol might be mediated via the activation of TRPM8-expressing innocuous cold receptors, assuming that the receptors access central neurons. Although the precise mechanisms underpinning OPN are still uncertain, this study may serve as an entry point in furthering the mechanistic understanding of OPN. Oxaliplatin has also been shown to modify intracellular Ca^2+^ handling within the cell bodies of cultured neurons (Grolleau et al. 2001). A more recent study cited a possible mechanism for some of the oxaliplatin-induced effects that is related to the modification of surface charges around the ion channel: either due to extracellular Ca^2+^ chelation or binding of a charged biotransformation product of oxaliplatin to the channel (Broomand et al. 2009). In addition, the prospective CONcePT study confirmed that OPN could be strongly attenuated by pre- and post-treating patients with Ca^2+^ and Mg^2+^ infusions (Gamelin et al. 2008). These findings suggest a mode of action that involves a Ca^2+^-dependent mechanism in OPN. Therefore, the Ca^2+^ ion channel TRPM8 appears to be a good candidate for understanding the Ca^2+^-dependent mechanism in OPN.

The TRP ion channel family consists of approximately 28 mammalian cation channels (Gaudet, 2008; Talavera et al. 2008; Eid and Cortright, 2009) that are involved in a wide range of physiological and pathophysiological processes including taste, thermosensation, pain, and cell cycle regulation. The TRP ion channels present a novel mechanism for controlling Ca^2+^ transients in human neurons and represent potential targets for regulating neurite proliferation and outgrowth. Recent studies have shown that regulating TRPM8 ion channels may be a way of controlling Ca^2+^ transients in human neurons. We, therefore, hypothesized that oxaliplatin could alter calcium-sensitive voltage-gated Na channels through a pathway that involves Ca^2+^ ions that are likely mobilized by TRPM8.

Several limitations should be considered in light of our results. Firstly, we did not conduct additional follow-up of CDT after oxaliplatin infusion. Such data would provide a context for the length of time it takes for the CDTs to return to normal and would be very useful from a clinical translation standpoint to approximate the outcome of patients after oxaliplatin infusion. This approach will be incorporated into our next protocol. Second, we compared our CDT findings against the CTCAE grading system that is a gross general measure of neuropathy impairment (Trotti et al. 2003) that precludes specific measurement of cold allodynia symptoms. Hence, our menthol testing needs validation against a testing method that provides an objective evaluation of cold allodynia/parasthesia, preferably the gold standard of CS, such as quantitative sensory testing. The validation of the menthol testing using quantitative sensory tests will be one of the important future studies. In addition, although our healthy subjects and chemotherapy-naïve patients were similar in age, sex, and baseline CDTs, having colon cancer patients as controls rather than healthy volunteers would have established equivalency at baseline by accounting for the potential influence of cancer-specific changes on CDTs. Future studies would benefit from conducting additional evaluations of CDTs after oxaliplatin infusion, performing quantitative sensory testing, and using patients with colon cancer without OPN as controls.

The present data show that menthol may be used to determine and evaluate the neurotoxicity severity score, although the methodology using menthol has not been firmly established. Interestingly, patients with prior oxaliplatin exposure had significantly elevated CDT at baseline, and patients with grade 3 neurotoxicity did not show significant changes in the CDT before and after oxaliplatin administration. These findings suggest that TRM8 may be associated with the chronic stage of OPN. Unfortunately, in this study, these patients were not prospectively monitored for changes in the CDT during and after a long period of oxaliplatin treatment therefore, we could not confirm whether or not the CDT increased with OPN progression. A prospective, multicenter, randomized, double-blind study is needed to investigate the possibility of CDT as a diagnostic marker for OPN.

In conclusion, our findings indicate that OPN may be associated with TRPM8 in acute hypersensitivity to CS, and that additional studies on TRPM8 will enhance our understanding of the mechanisms of OPN. Further, our study demonstrates the feasibility of undertaking CDT test in a clinical setting to facilitate the identification of early neurotoxicity, although larger trials need to be conducted to confirm our findings.
